# Fully-automated estimation of upper cervical cord cross-sectional area using pontomedullary junction referencing in multiple sclerosis

**DOI:** 10.3389/fnimg.2025.1681669

**Published:** 2025-11-04

**Authors:** Roberto Masciullo, Annine Sutter, Rosaria Sacco, Nicola Pinna, Daniela Distefano, Emanuele Pravatà, Giulia Mallucci, Alessandro Cianfoni, Claudio Gobbi, Chiara Zecca, Giulio Disanto

**Affiliations:** ^1^Department of Neuroradiology, Neurocenter of Southern Switzerland, Ente Ospedaliero Cantonale, Lugano, Switzerland; ^2^Multiple Sclerosis Center, Neurocenter of Southern Switzerland, Ente Ospedaliero Cantonale, Lugano, Switzerland; ^3^Faculty of Biomedical Sciences, Università della Svizzera Italiana, Lugano, Switzerland; ^4^Department of Neuroscience, Imaging and Clinical Sciences, Università degli studi “G. d’Annunzio” Chieti-Pescara, Chieti, Italy

**Keywords:** spinal cord, cross-sectional area, deep-learning, pontomedullary junction, multiple sclerosis

## Abstract

**Background:**

Spinal cord cross-sectional area (CSA) is a biomarker of disability in multiple sclerosis (MS). Vertebral-based CSA suffers from anatomical variability and positional bias.

**Objectives:**

To evaluate a fully automated PMJ-referenced approach, as implemented in the open-source Spinal Cord Toolbox, to assess cervical cord CSA at a fixed distance from the pontomedullary junction (PMJ) in MS.

**Methods:**

Retrospective study performed at the MS center of Lugano (Switzerland). Inclusion criteria were treatment with natalizumab or ocrelizumab and absence of clinical/radiological disease activity over ≥2 years. CSA at 64 mm caudal to the PMJ (CSA PMJ) and at C2–C3 vertebral level (CSA C2–C3) were calculated using the Spinal Cord Toolbox.

**Results:**

Seventy-five MS patients [females = 44 (58.7%), age = 45.1 (36.7–53.8) years, natalizumab = 36 (48%), ocrelizumab = 39 (52%)] were included. Median CSA PMJ and CSA C2–C3 were 57.7 (53.1–62.1) and 58.1 (53.2–62.6) mm^2^, respectively. The two measures were highly correlated (rho = 0.95, *p* < 0.001), with some exceptions related to errors in vertebral labelling in CSA C2–C3 assessments. PMJ was correctly identified in all subjects. CSA PMJ measures were negatively associated with disability (*β* = −0.08, *p* = 0.002), independent of age and sex.

**Conclusion:**

Automated measurement of spinal cord CSA at fixed distance from the PMJ is applicable in MS, performs better than vertebral-based CSA, and correlates with neurological disability.

## Introduction

Spinal cord atrophy is a well-established imaging biomarker of neurodegeneration in multiple sclerosis (MS), and is strongly associated with neurological disability (Losseff et al., 1996; Rocca et al., 2011). Among several metrics, the cross-sectional area (CSA) of the upper cervical cord typically measured at the C2–C3 intervertebral disc level has emerged as a reproducible, sensitive indicator of spinal cord damage (Lukas et al., 2021). However, vertebral-based referencing is sensitive to neck flexion/extension, varying slice angulation, and labelling variability, limiting inter-subject comparability and longitudinal precision (Cohen-Adad et al., 2021; Valošek and Cohen-Adad, 2024). In addition, the variable correspondence between vertebral levels and spinal cord segmental levels further increases inter-subject variability (Cadotte et al., 2015).

Methodological advances have shifted attention toward intrinsic anatomical landmarks, notably the pontomedullary junction (PMJ) to provide a more robust spatial referencing for CSA measurements (Bédard et al., 2023). Bédard and Cohen-Adad (2022) have introduced a framework for computing CSA at a fixed distance caudal to the PMJ, reducing sensitivity to variable cord curvature. Automated spinal cord segmentation and vertebral labelling have also matured substantially with the introduction of deep learning methods and robust template-based approaches (Bédard and Cohen-Adad, 2022; Gros et al., 2019; Keegan et al., 2024). These have been recently integrated into the open-source Spinal Cord Toolbox (SCT), which allows scalable and reproducible CSA quantification at both C2–C3 and at a fixed distance from the PMJ, requiring minimal user input and supporting large cohort analyses (Bédard and Cohen-Adad, 2022).

As an additional source of variability, there is no universal agreement regarding how CSA measurements should be normalized, if by brain volume, intracranial volume and/or spinal canal area as potential alternatives (Keegan et al., 2024). The SCT currently provides a normalization method that is based on sex and brain volume measures, collected from a large cohort of individuals without a history of neurological diseases (Bédard and Cohen-Adad, 2022). However, this may be problematic in patients suffering from neurological conditions which also cause brain atrophy, as this would bias normalized CSA estimates (Bédard and Cohen-Adad, 2022; Keegan et al., 2024).

In this study, we evaluated the benefits of a fully automated pipeline for upper cervical cord CSA calculation in patients with MS using PMJ referencing, in terms of applicability, relation to C2–C3-based CSA measurements, and association with disability scores. We also aimed to compare raw CSA measurements to those normalized based on brain and intracranial volumes in terms of association with disability scores.

## Materials and methods

### Patient population and setting of the study

This was a single center retrospective study conducted at the MS center of the Neurocenter of Southern Switzerland (Lugano, Switzerland). All patients at our center are routinely followed-up every approximately 3 months, with neurological examinations and expanded disability status scale (EDSS) estimations performed by certified raters (Kurtzke, 1983). Brain and upper spinal cord MRIs are performed in all patients at least once a year and in case of suspected relapses.

From the overall population of MS patients, those fulfilling the following inclusion criteria were consecutively recruited: (1) A diagnosis of relapsing–remitting (RR), secondary progressive (SP) or primary progressive (PP) MS by the 2017 revision of McDonald criteria (Thompson et al., 2018); (2) Age above 18 years; (3) Being treated with either natalizumab (NTZ) or ocrelizumab (OCR) for ≥2 years; (4) Absence of new clinical relapses and absence of new T2 demyelinating lesions on brain and spinal MRI over the last 2 years of treatment; (5) Having performed a high-quality brain and upper cord MRI (see below). Exclusion criteria were: (1) Pregnancy; (2) Inability to follow procedures or insufficient knowledge of project language; (3) Inability to provide consent.

### MRI studies and image processing

After inclusion in the study, the last MRI performed by the patients was retrospectively selected for analysis. All MRIs were performed using a Siemens Skyra 3T scanner with a standardized acquisition protocol based on the recommendations from Cohen-Adad et al. (2021) for quantitative spinal cord MRI. A 3D T1-weighted magnetization prepared rapid acquisition (MPRAGE) sequence, ensuring appropriate coverage of the brainstem and upper cervical spinal cord down to the C4 level, was employed. The sequence was acquired with the following parameters: voxel size = 1 × 1 × 1 mm^3^, TR = 2,300 ms, TE = 2.98 ms, TI = 900 ms, and Field of View = 256 × 256 mm.

Images were processed using the SCT, version 6.5, an open-source software specifically developed for spinal cord MRI analysis (De Leener et al., 2017). Segmentation of the spinal cord was carried out automatically using the deep learning-based algorithm sct_deepseg_sc, which has demonstrated robust accuracy across multiple contrasts and clinical populations (Gros et al., 2019). The PMJ was identified automatically using the sct_detect_pmj function, and the CSA was calculated at a fixed distance of 64 mm caudal to this landmark computed along the spinal cord centerline (geodesic distance) and then averaged on a 20 mm extent (CSA PMJ). The CSA computation was performed in each participant’s native space, using orthogonal slices with respect to the cord centerline, in order to account for its curvature as previously proposed (Bédard and Cohen-Adad, 2022; Gros et al., 2019). CSA was also measured at the conventional C2–C3 intervertebral disc level (CSA C2–C3), with vertebral labelling performed using the SCT command sct_label_vertebrae (Ullmann et al., 2014).

All scripts and parameters files used for the SCT workflow are available on GitHub,^1^ and also in the SCT website.^2^ We checked the results of the SC segmentations and of the PMJ and vertebral labelling using SCT’s quality report (sct_qc).

In order to minimize inter-subject variability due to anatomical differences, spinal cord CSA values were normalized using a regression-based method, i.e., the -normalize function in SCT, by both brain and intracranial volumetric data. Total brain volume (BV) was computed from 3D T1-weighted images using SIENAX (part of FSL) (Smith et al., 2004; Puonti et al., 2016). Intracranial volume (IV, i.e., total volume inside the skull, including brain tissue and cerebrospinal fluid) was independently estimated using SAMSEG (Sequence Adaptive Multimodal SEGmentation) from FreeSurfer v. 8.0.0^3^ (Puonti et al., 2016).

### Statistical analyses

Categorical variables were described by counts and percentages, continuous and ordinal variables by median, interquartile range (IQR) and range. Pairwise correlations were tested after assessment of normality (by histograms and Shapiro–Wilk test) using the Pearson’s correlation coefficient. Multivariate linear regression models were used to test CSA measures for association with disability scores (EDSS), adjusted by age and sex. Linear assumptions were checked using residuals vs. predicted plots and qqplots. The performance of different regression models was compared using R-squared estimates (i.e., the proportion of variance in the dependent variable that can be explained by the independent variables) and Akaike Information Criterion (AIC, mathematical estimate of how well a model fits the data, based on number of independent variables and maximum likelihood estimate of the model). All statistical analyses were conducted using R (version 4.4.2) and the R packages “DHARMa” and “AICcmodavg.”

## Results

A total of 75 MS patients were included in the study (Table 1). The median age at the time of MRI acquisition was 45.1 (36.7–53.8) years, and 44 patients were female (58.7%). Thirty-six (48%) patients were on treatment with NTZ, 39 (52%) with OCR. Only two patients had progressive MS. Median EDSS was 2.5 (1.5–3.5), with scores ranging between 0 and 7.

**Table 1:** Baseline demographic and clinical characteristics of patients included in the study.

Variable		Median/count	IQR (%)	Range
Age at MRI (years)		45.1	36.7–53.8	18.2–74.0
Sex	F	44	58.7	
	M	31	41.3	
DMT	NTZ	36	48	
	OCR	39	52	
Disease course	RRMS	73	97.3	
	PMS	2	2.7	
Time between MRI and EDSS (years)		0.1	−0.1 to 0.4	−1 to 1
EDSS		2.5	1.5–3.5	0–7

EDSS, expanded disability status scale; IQR, interquartile range; MRI, magnetic resonance imaging; NTZ, natalizumab; OCR, ocrelizumab; PMS, progressive MS; RRMS, relapsing-remitting multiple sclerosis.

### Raw CSA PMJ vs. CSA C2–C3

**Figure 1:**
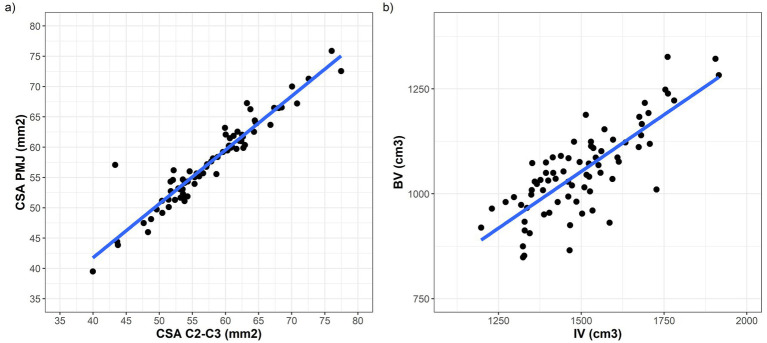
**(a)** Strict correlation between CSA measures estimated at C2–C3 vertebral level (*x* axis) and using the PMJ referenced method (*y* axis). **(b)** Correlation between intracranial volume (on the *x* axis) and raw brain volume (on the *y* axis).

We first estimated the raw (i.e., not normalized) CSA PMJ and CSA C2–C3 measures, and how these correlated to each other. The median CSA PMJ was 57.7 (IQR = 53.1–62.1, range = 39.5–75.9) mm^2^. The median CSA C2–C3 was 58.1 (IQR = 53.2–62.6, range = 39.9–77.5) mm^2^. There was a strong overall correlation between the two measures (Pearson’s rho = 0.95, 95% CI = 0.92–0.97, *p* < 0.001, see Figure 1a).

**Figure 2:**
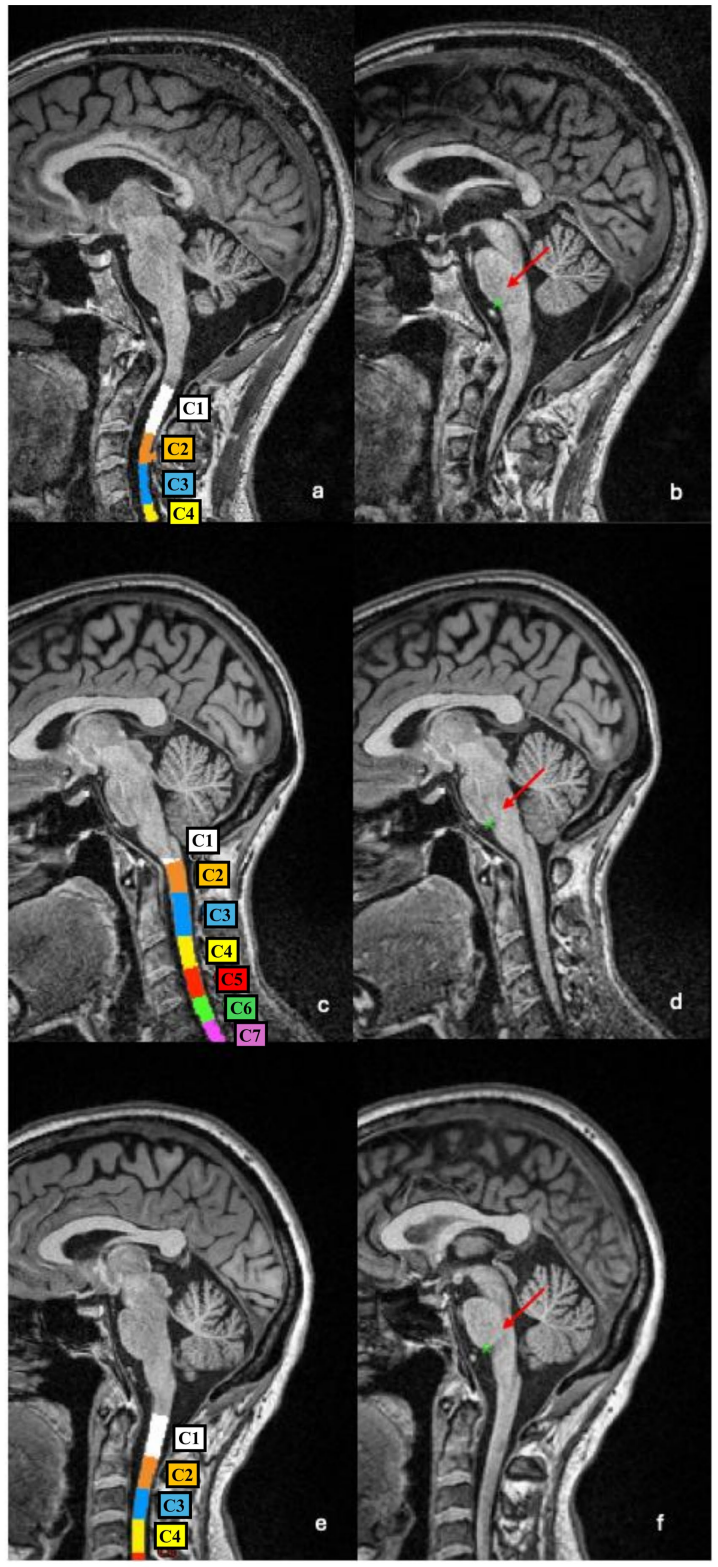
Examples of vertebral segmentation and labelling errors. **(a,b)** In this patient the C1–C2 complex and C3 vertebras were incorrectly labelled as C1 and C2, respectively, resulting in a downward shift of all subsequent levels **(a)**. As a consequence, the CSA measured at the (misidentified) C2–C3 level is underestimated (43.32 mm^2^ vs. 55.99 mm^2^ if vertebral labelling is manually corrected). The PMJ-referenced segmentation method correctly detects the PMJ **(b)**, and the CSA measured at 64 mm caudal to the PMJ is more anatomically accurate (57.09 mm^2^). **(c,d)** In this patient, part of the C2 vertebral body was incorrectly labelled as C3 **(c)**, leading to an overestimation of CSA (77.46 mm^2^ vs. 71.68 mm^2^ if vertebral labelling is manually corrected). The PMJ identification is accurate **(d)**, and the CSA measured 64 mm caudal to the PMJ appears more reliable (72.56 mm^2^). **(e,f)** In this patient, the vertebral labelling is anatomically correct **(e)** as well as the identification of the PMJ **(f)**. CSA C2–C3 and CSA PMJ measures are therefore more comparable (55.33 mm^2^ and 55.05 mm^2^, respectively).

Despite the observed strong correlation, some notable discrepancies emerged in a subset of patients. Upon visual inspection of the segmentations and vertebral labelling, we identified that the PMJ was consistently and accurately detected across all subjects (75/75, 100%), while the vertebral labelling algorithm misidentified the C2–C3 disc level in 24/75 (32%) subjects. Figure 2 shows examples of vertebral labelling that are, respectively, incorrect, partially inaccurate, and anatomically correct at the C2–C3 level, and how this affected CSA C2–C3 measures. In contrast, the PMJ was accurately and automatically identified in all patients.

### Effect of normalization methods on CSA PMJ

To understand how normalization affects CSA PMJ measurements, we calculated the raw BV and IV for each subject. The median BV and IV were 1,045 (980.8–1,112.6) and 1,485 (1,386–1,590) cm^3^, respectively (Supplementary Figure S1; Supplementary Table S1). As expected, IV was always larger than BV [median delta 448.7 (368.6–510.7) cm^3^], with a strong correlation between these two measures (Pearson’s rho = 0.79, 95% CI = 0.68–0.86, *p* < 0.001; Figure 1b).

**Figure 3:**
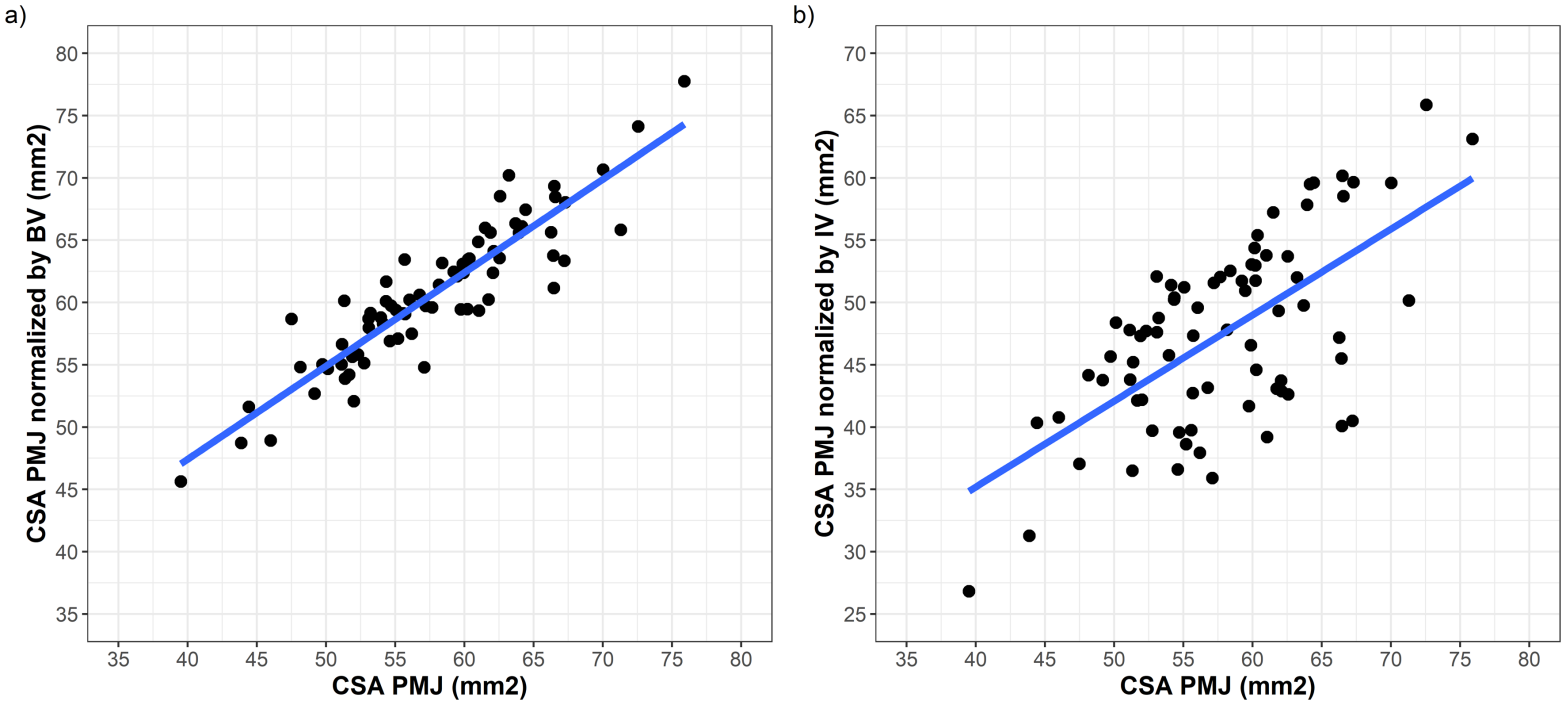
Scatter plots showing the correlation between raw CSA PMJ on the *x* axis and CSA PMJ measurements normalized by BV **(a)** and IV **(b)**.

**Figure 4:**
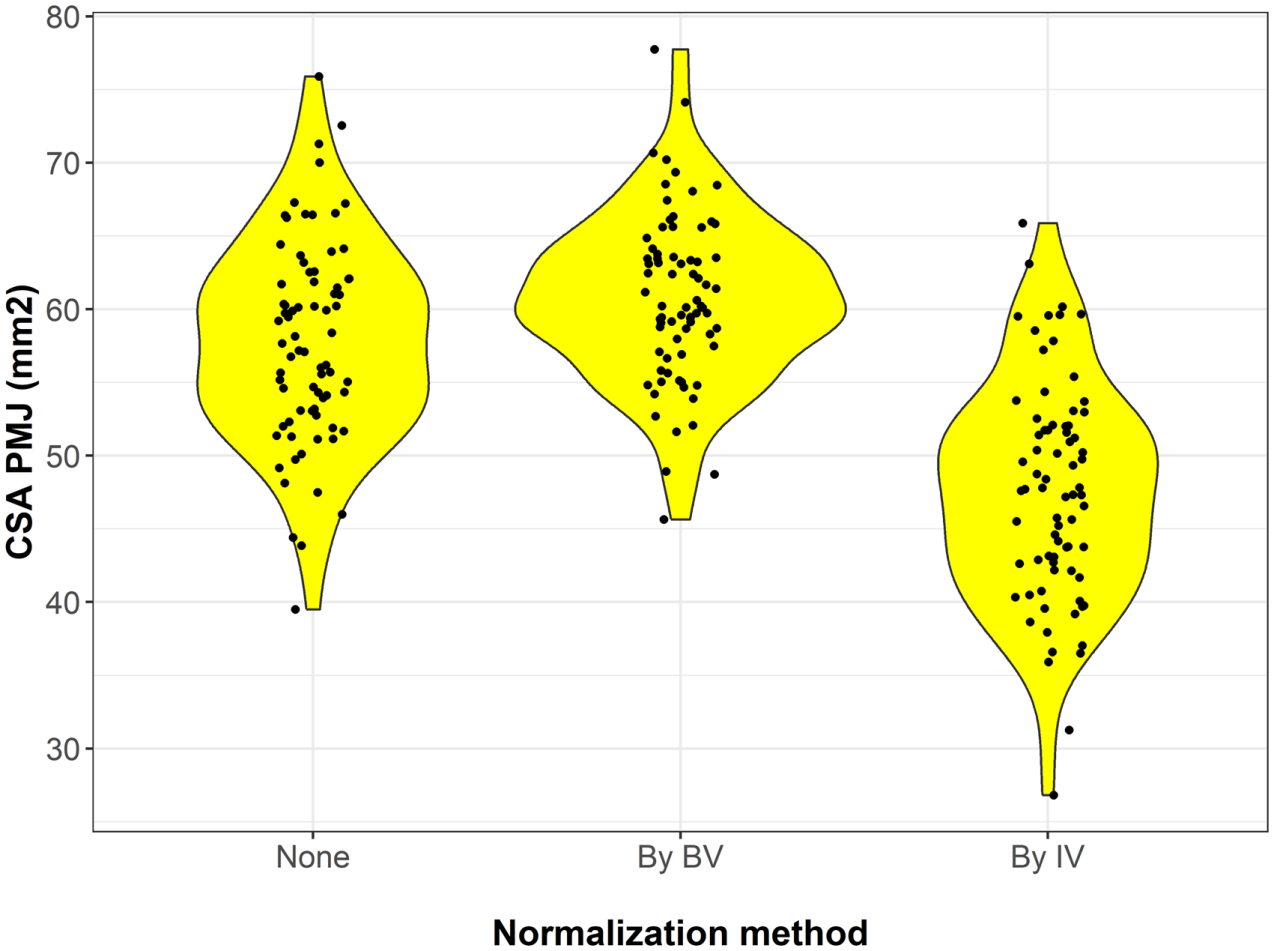
Violin plots showing the distribution of raw CSA PMJ value (on the left) and those normalized by BV and IV.

Both normalization methods produced CSA PMJ measurements that were highly correlated with raw CSA PMJ (normalized by BV: Pearson’s rho = 0.91, 95% CI = 0.86–0.94, *p* < 0.001; normalized by IV: Pearson’s rho = 0.64, 95% CI = 0.48–0.75, *p* < 0.001; Figure 3). However, normalization of CSA PMJ had a substantial impact on both the magnitude and distribution of the measurements, especially when normalized by IV (Figure 4). As compared to raw CSA PMJ, the CSA PMJ normalized by BV was increased by a median of 3.2 (1.9–4.7) mm^2^, whereas the CSA PMJ normalized by IV was decreased by a median of −8.4 (−14.4 to −5.4) mm^2^ (Supplementary Table S1).

### Association between CSA PMJ and disability scores

**Figure 5:**
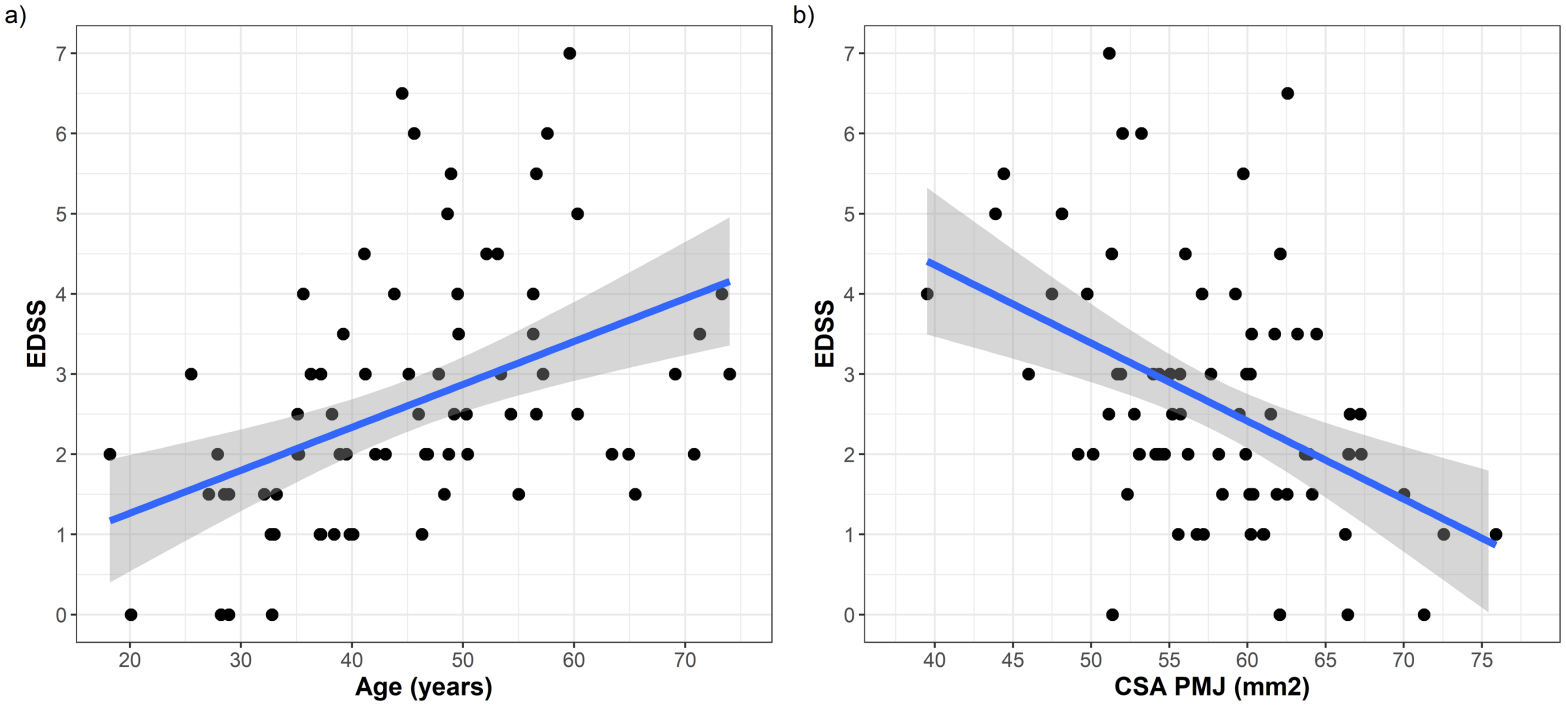
**(a)** Scatter plot showing the positive association between age (on the *x* axis) and EDSS (on *y* axis); **(b)** Scatter plot showing the negative association between raw CSA PMJ (on the *x* axis) and EDSS (on *y* axis).

We next assessed the relationship between CSA PMJ and clinical disability using a series of linear regression models adjusted for age and sex (Table 2). As expected, age was positively associated with EDSS scores in all models (Figure 5a; Table 2). Raw CSA PMJ also showed a significant inverse association with EDSS (*β* = −0.08, *p* = 0.002) and this was independent of age and sex (Figure 5b; Table 2). When comparing different normalization strategies, all CSA PMJ normalized measures showed significant associations with EDSS, but this appeared stronger for CSA PMJ normalized by IV than by BV. The model including CSA PMJ normalized by IV was the one showing the greatest R-squared and smallest AIC (indicating better performance in explaining EDSS), followed by raw CSA PMJ and then by BV (Table 2). Raw CSA PMJ remained associated with EDSS (*β* = −0.06 *p* = 0.029), also when BV was included as an additional covariate in the regression model.

**Table 2:** Multivariate linear regression models testing the association between CSA PMJ (raw, normalized by BV and by IV) with EDSS scores.

Linear regression models predicting EDSS		*β*	*p* value	R-squared	AIC
Sex	F	–	–	0.300	261.4
	M	0.00	0.992		
Age (per year)		0.04	0.001		
Raw CSA PMJ (per mm^2^)		−0.08	0.002		
Sex	F	–	–	0.268	264.74
	M	−0.11	0.729		
Age (per year)		0.05	<0.001		
CSA PMJ normalized by BV (per mm^2^)		−0.07	0.010		
Sex	F	–	–	0.315	259.77
	M	−0.93	0.015		
Age (per year)		0.04	<0.001		
CSA PMJ normalized by IV (per mm^2^)		−0.09	<0.001		

All models are adjusted by age and sex. Regression metrics (R-squared and AIC) are shown for each model on the right side of the table. AIC, akaike information criterion; BV, brain volume; EDSS, expanded disability status scale; IV, intracranial volume.

## Discussion

We investigated the applicability of a fully automated pipeline for measuring spinal cord CSA at a fixed distance caudal to the PMJ in patients with MS. To the best of our knowledge, this is the first study to apply this PMJ-referenced approach combined with deep learning segmentation and volumetric normalization in a clinical MS cohort. Our findings demonstrate the CSA PMJ not only outperforms the conventional vertebral-based CSA estimation at C2–C3 level, but also shows robust associations with neurological disability. Notably, the CSA PMJ of our patients was on average smaller than that of the general population measured in the original publication implementing this method (mean CSA = 66.2 mm^2^ ± 6.7, range 51.9–95.6) (Bédard and Cohen-Adad, 2022). Despite the differences in settings and population, this may be at least in part related to the extent of spinal atrophy in MS.

One of the key strengths of the PMJ-referenced method was its anatomical consistency. Unlike vertebral-level labelling, which is susceptible to variability due to neck position, image angulation, and inter-individual anatomical differences, the PMJ landmark was reliably and automatically identified across patients. Visual inspection confirmed that while vertebral labelling errors were present in several cases leading to over- or under-estimation of CSA, automated PMJ identification remained accurate. Compared with vertebral-level referencing, PMJ-referenced sampling therefore yielded more accurate anatomical localization, and required fewer manual interventions. This robustness supports its use as a reliable reference point for spinal cord morphometry, particularly in the settings of multicenter studies in which standardized analyses may be needed to be applied to a large number of individuals. This and other similar automated methods also have the potential to provide support to radiologists and neurologists in clinical settings, particularly in the monitoring of atrophy rates over time (Collorone et al., 2024).

Normalization of spinal cord CSA estimates are particularly relevant for cross-sectional studies. Currently, there is no uniform agreement on how CSA measures should be normalized (Keegan et al., 2024; Papinutto et al., 2020). Brain volume, intracranial volume and spinal canal area have all been proposed as potential normalization parameters (Papinutto et al., 2020; Kesenheimer et al., 2021; Nigri et al., 2023). In MS studies, the volumetric scale factor estimated by SIENAX has also been often used to normalize spinal cord CSA, as an indirect measure of intracranial volume (Rocca et al., 2023; Bischof et al., 2022; Fein et al., 2004). Notably, the SCT method developed by Bédard and Cohen-Adad (2022) was designed to normalize spinal cord CSA by sex and brain volume (or alternatively thalamic volume). As stated in their original publication, applying this method to individuals affected by a condition that is also causing brain/thalamic volume loss, as in the case of MS, can generate biased CSA estimates. Indeed, any CSA measure should be theoretically normalized using a pre-morbid BV measure. This is often problematic in MS patients, who typically show higher rates of brain atrophy compared to the general population since the initial stages of disease (Fein et al., 2004; Rojas et al., 2015; Azevedo et al., 2015).

Having said this, the association between CSA PMJ and EDSS was of weaker magnitude when CSA measures were normalized by BV, as compared to raw CSA PMJ. We interpret this as a consequence of the extent of BV loss in this sample of patients, which limits the use of BV as a normalization parameter. Therefore, while normalization of CSA measures for BV may be useful for reducing inter-subject anatomical variability, this may introduce a relevant bias in CSA estimates in the specific setting of MS. We next tested whether normalizing the CSA PMJ by IV could represent an option, since this would not be affected by pathological brain volume loss. To do this, we directly calculated the IV rather than using the SIENAX scaling factor as an indirect measure of this. Interestingly, the regression model testing IV-normalized CSA PMJ measures for association with EDSS had the best predictive performance. There is a known relation between IV and BV in the general population, as also seen in our own cohort (Wang et al., 2024). While this may suggest the possibility to apply the same normalization procedure based on BV to IV, it is important to remember that the method developed by Bédard and Cohen-Adad (2022) was not built on IV, and further studies would be needed to develop a normalization procedure that is based on IV. We did not normalize CSA by spinal canal metrics because dedicated axial acquisitions for reliable canal area estimation were not available for all subjects, and also for the lack of a reliable automatic estimation of the spinal canal segmentation. Canal-based normalization can reduce inter-subject anatomical variance and may be advantageous when brain measures are influenced by the disease, and incorporating robust canal metrics could improve future studies.

This study has several strengths. First, this is the first report investigating automated AI-based measurement of CSA PMJ in MS. Second, we investigated a relatively homogenous sample of MS patients, all on treatment with high efficacy therapies, and no inflammatory clinical or radiological activity at the time of the study. This reduces the potential risk of confounding by different therapies and concurrent inflammatory disease activity. Third, all patients performed standardized neurological examinations and MRI protocols on identical 3T MRI scans. Last, we were able to integrate brain and intracranial volume metrics in the normalization process of spinal cord CSA.

There are also several limitations. CSA inherently depends on the accuracy of cord segmentation. Small boundary errors (under-/over-segmentation or partial inclusion of nerve rootlets) can propagate to area estimates and inflate within-subject variability, particularly at levels affected by motion or cerebrospinal fluid pulsation. The software computed CSA on slices orthogonal to the local centerline and averaged across contiguous slices to reduce local noise. Nevertheless, residual segmentation errors may persist and would affect both PMJ-referenced and vertebral-referenced measures. Future work should incorporate automated QC metrics (e.g., outlier detection on perimeter/area and centerline smoothness), uncertainty estimates from the segmentation model, and formal scan–rescan experiments to quantify segmentation-driven variance.

Sampling CSA at a fixed caudal distance from the PMJ assumes limited inter-individual variability in cervical cord length, which represents an additional limitation. We did not have a control sample of individuals to compare CSA PMJ values against those collected from MS patients, and the retrospective single-center design may limit generalizability to other MS populations. It would be important to confirm these results in larger independent samples of patients. We did not include scan–rescan test–retest reproducibility. Prior PMJ-referenced work in healthy participants reported low within-session variability across neck positions, supporting short-term robustness to posture changes. However, formal between-session test–retest especially in MS cohorts remains to be established (Bédard et al., 2023). Finally, the pipeline assumes availability of high-quality 3D T1-weighted images, which may not be routinely acquired in all clinical settings.

To conclude, our findings support the clinical validity of PMJ-referenced spinal cord CSA as a biomarker in MS. The method developed by SCT is fully automated, reproducible, and robust to anatomical variability introduced by vertebral labelling. Raw CSA PMJ values were significantly associated with neurological disability. Further work is needed to optimize normalization strategies that take into account the potential degree of brain atrophy in MS, and this is particularly relevant for cross-sectional applications. The methodology appears instead already compatible with longitudinal applications and could serve as a standardized approach for tracking spinal cord atrophy over time. Given its precision and scalability, the PMJ-based CSA measurement has the potential to become a standardized tool in both clinical and research neuroimaging settings in MS.

## Data Availability

The raw data supporting the conclusions of this article will be made available by the authors, without undue reservation.

## Footnotes

^1^ https://github.com/sct-pipeline

^2^ https://spinalcordtoolbox.com

^3^ https://surfer.nmr.mgh.harvard.edu
