# Supplementary material for: Fully-automated estimation of upper cervical cord cross-sectional area using pontomedullary junction referencing in multiple sclerosis
Source: Front Neuroimaging. 2025 Nov 4;4:1681669. doi: 10.3389/fnimg.2025.1681669 (PMC12623166; doi:10.3389/fnimg.2025.1681669)

**SUPPLEMENTARY MATERIAL**

**Supplementary table 1:** Volumetric estimates of crude CSA PMJ, CSA PMJ normalized by brain volume (BV), CSA PMJ normalized by IV, as well as BV and IV.

| **Variable** | **Median** | **IQR** | **Range** |
| --- | --- | --- | --- |
| **Crude CSA PMJ (mm2)** | 57.7 | 53.1 - 62.1 | 39.5 - 75.9 |
| **CSA PMJ normalized by BV (mm2)** | 60.2 | 57.3 - 63.7 | 45.6 - 77.8 |
| **CSA PMJ normalized by IV (mm2)** | 47.6 | 42.4 - 52.0 | 26.8 - 65.9 |
| **BV (cm3)** | 1045 | 980.8 - 1112.6 | 848.6 - 1326.3 |
| **IV (cm3)** | 1485 | 1386 - 1590 | 1197 - 1915 |
| **IV - BV delta (cm3)** | 448.7 | 368.6 - 510.7 | 265.2 - 716.8 |

**Supplementary figure 1:** Violin plots showing distribution of brain volume and intracranial volume.


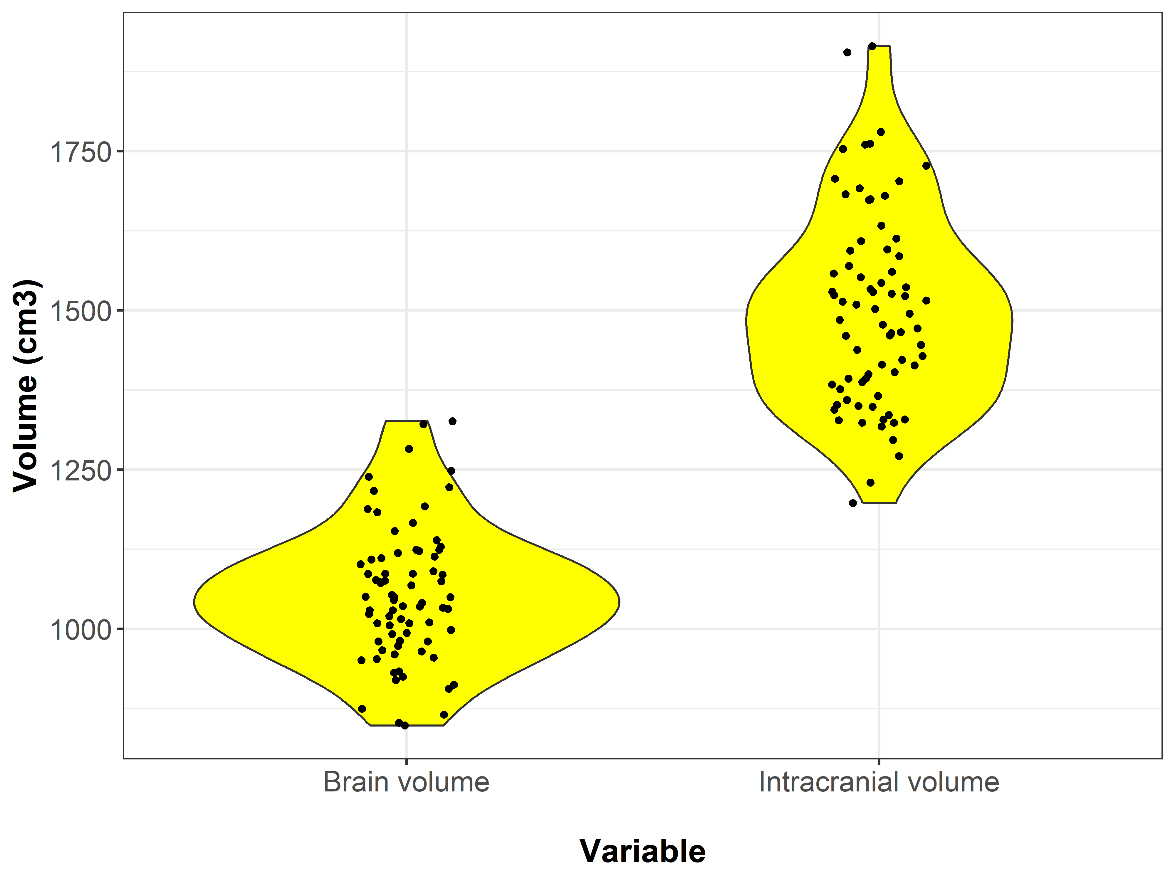

Supplement: Supplementary file 1 [file Table_1.DOCX]
